# Homage to Richard M. Elliott

**DOI:** 10.3390/v8080224

**Published:** 2016-08-10

**Authors:** Alain Kohl, Benjamin Brennan, Friedemann Weber

**Affiliations:** 1 MRC-University of Glasgow Centre for Virus Research, Glasgow G61 1QH, Scotland, UK; alain.kohl@glasgow.ac.uk (A.K.); Ben.Brennan@glasgow.ac.uk (B.B.); 2 Institute for Virology, FB10—Veterinary Medicine, Justus-Liebig University, Gießen 35392, Germany

In the last 25 years, the scientific and public attention paid to bunyaviruses has increased considerably. This has many reasons (one of them being that new family members are constantly emerging) and many drivers, but there was one man whose name will be forever connected with the *Bunyaviridae* family. Richard M. Elliott passed away in 2015 at the age of 61. With his unstoppable enthusiasm, strong vision, perseverance, and his keen interest in almost every aspect of bunyavirus replication, he greatly contributed to the progress in the field [1]. While his most prominent achievement may be the first rescue of a segmented negative-strand RNA virus (the type species Bunyamwera) from plasmid cDNA, he also studied glycoprotein processing, particle assembly, anti-interferon mechanisms, phylogeny, vaccine development and host cell factors. Yes, these are the main topics of this special issue of Viruses, and Richard could have chosen any of them to write a competent review himself. Lamentably, this is not possible any more, but we will always remember the man who helped to foster so much of the past research and train the next generation of scientists studying the “Cinderellas of virology” (in his own words) which in reality constitute one of the biggest virus families ever known.
